# Predictors for major cardiovascular outcomes in stable ischaemic heart disease (PREMAC): statistical analysis plan for data originating from the CLARICOR (clarithromycin for patients with stable coronary heart disease) trial

**DOI:** 10.1186/s41512-017-0009-y

**Published:** 2017-03-29

**Authors:** Per Winkel, Janus Christian Jakobsen, Jørgen Hilden, Theis Lange, Gorm Boje Jensen, Erik Kjøller, Ahmad Sajadieh, Jens Kastrup, Hans Jørn Kolmos, Anders Larsson, Johan Ärnlöv, Christian Gluud

**Affiliations:** 1 Copenhagen Trial Unit, Centre for Clinical Intervention Research, Department 7812, Blegdamsvej 9, Rigshospitalet, Copenhagen University Hospital, Copenhagen, Denmark; 2 Department of Cardiology, Holbæk Hospital, Holbæk, Denmark; 3 Section of Biostatistics, Department of Public Health, University of Copenhagen, Copenhagen, Denmark; 4 Department of Cardiology, Hvidovre Hospital, Copenhagen University Hospital, Copenhagen, Denmark; 5 Department of Cardiology S, Herlev Hospital, Copenhagen University Hospital, Copenhagen, Denmark; 6 Department of Cardiology, Bispebjerg Hospital, Copenhagen University Hospital, Copenhagen, Denmark; 7 Department of Cardiology B, The Heart Centre, Rigshospitalet, Copenhagen University Hospital, Copenhagen, Denmark; 8 Department of Clinical Microbiology, Odense University Hospital, Odense, Denmark; 9 Department of Neurobiology, Care Sciences and Society/Division of Family Medicine, Karolinska Institute, Stockholm, Sweden; 10 Department of Health and Social Sciences, Dalarna University, Falun, Sweden; 11 Center for Statistical Science, Peking University, Beijing, China

**Keywords:** CLARICOR, Ischaemic heart disease, Predictors, Biomarkers, Mortality

## Abstract

**Background:**

The purpose of the predictors for major cardiovascular outcomes in stable ischaemic heart disease (PREMAC) study is exploratory and hypothesis generating. We want to identify biochemical quantities which—conditionally on the values of available standard demographic, anamnestic, and biochemical data—may improve the prediction of cardiovascular outcomes and/or death in patients suffering from stable ischaemic heart disease. The candidate biochemical quantities include N-terminal pro-B-type natriuretic peptide, YKL-40, osteoprotegerin, high-sensitive assay cardiac troponin T (hs-cTnT), pregnancy-associated plasma protein-A (PAPP-A), cathepsin B, cathepsin S, soluble TNF receptor 1 and 2, neutrophil gelatinase-associated lipocalin, endostatin, and calprotectin. As an extra objective, we also want to assess if skewness in these predictors may explain why the clarithromycin for patients with stable coronary heart disease (CLARICOR) trial found increased all-cause and cardiovascular (CV) mortality on a brief clarithromycin regimen compared with placebo.

**Methods:**

Baseline data were obtained from the hospital files at five cardiology clinics covering the Copenhagen area. The CLARICOR trial included data from 4372 stable coronary artery disease patients recruited among such patients alive and diagnosed with acute myocardial infarction or unstable angina pectoris during 1993 to 1999 in Copenhagen and randomised during October 1999 to April 2000 to the CLARICOR trial of 14 days clarithromycin versus placebo.

Initial follow-up lasted for 2.6 years, during which outcomes were collected through hospital and death registries and assessed by an adjudication committee. Corresponding register data later showed to produce similar results. The adjudicated outcomes were therefore replaced and augmented by register data on outcomes to cover 10 years of follow-up. Biochemical marker data were obtained from analysis of serum from the CLARICOR bio-bank collected at randomisation and stored at −80° C.

Using Cox proportional hazard method, we will identify among the candidate biochemical quantities those which are significant predictors when used alone and in combination with the standard predictors as defined in the present study.

**Discussion:**

Patients who became stable during the period 1993 to 1999 and died before October 1999 are missing. The data from the placebo patients are nevertheless useful to identify new prognostic biomarkers in patients with stable coronary artery disease, and data from both trial groups are useful to assess important potential skewness between randomised groups. However, due to the potential selection bias, we do not feel that it is advisable to try to rank identified biochemical predictors relative to each other nor to use the results for predictive purposes.

**Trial registration:**

ClinicalTrials.gov, NCT00121550

Date of registration 13 July 2005

Date of enrolment of first participant 12 October 1999

## Background

Cardiovascular diseases, and ischaemic heart disease in particular, affect large fractions of the elderly population and constitute one of the two dominant causes of death [1–5]. Identifying high-risk patients would allow one to assess more aggressive measures for treatment of cardiovascular disease [6]. The Prognosis Research Strategy (PROGRESS) series introduced a framework of four themes [7–9], including identification of specific factors (such as biomarkers and treatment modalities) that are associated with prognosis (prognostic factor research). This theme is the focus of the present “predictors for major cardiovascular outcomes in stable ischaemic heart disease” (PREMAC) study.

## Methods

### Objectives of the PREMAC study

Our primary objective is to identify biochemical predictors of a combined outcome including acute myocardial infarction (AMI), unstable angina pectoris (UAP), cerebro-vascular disease (CeVD), cardiovascular mortality (CV death), and all-cause mortality in non-hospitalised patients with stable coronary artery disease.

### The patient group and data material

The patient group comprises the patients who participated in the clarithromycin for patients with stable coronary heart disease (CLARICOR) trial [10, 11], and the data material includes the baseline data collected at randomisation augmented by biomarker data obtained by analysing the bio-bank material collected at baseline and outcome data prospectively obtained from public registers [12].

#### The patients

The patient group is defined by the inclusion and the exclusion criteria of the patients who, during the winter 1999–2000, entered the CLARICOR trial.

The CLARICOR trial was an investigator-initiated, randomised, placebo-controlled, multicentre superiority trial including outpatients with stable coronary artery disease (CAD), using central 1:1 randomisation and blinding of all parties. All patients discharged from wards or outpatient clinics in the Copenhagen area were available in an existing database. We invited all 13,702 patients who were alive and aged 18–85 years in 1999 and identified with a diagnosis of myocardial infarction or unstable angina pectoris during the years 1993–1999 to visit one of five cardiology centres in the Copenhagen area. Six thousand one hundred sixteen (44.6%) patients accepted the invitation, and of these, 4372 (71.5%) were randomised, while 1567 (25.6%) were excluded, and 177 (2.9%) refused to participate. Exclusion criteria included AMI or UAP within the previous 3 months, percutaneous transluminal coronary angioplasty and coronary bypass surgery within the previous 6 months, impaired renal or hepatic function, congestive heart failure (New York Heart Association (NYHA) IV classification of heart failure), active malignancy, incapacity to manage own affairs, breast feeding, and possible pregnancy. Between October 1999 and April 2000, the 4372 patients were randomised to receive oral clarithromycin 500 mg once daily for 2 weeks versus matching placebo to assess the effects on the risk of major cardiovascular outcomes and death.

The main results of the CLARICOR trial were that clarithromycin increased the risk of cardiovascular as well as all-cause mortality [12–14].

#### The data

Data material collected from the 4372 patients include (1) demographic and anamnestic (hospital) data gathered prior to the randomisation, (2) values of biochemical quantities measured in plasma specimens obtained from the patients at randomisation, and (3) vital data and diagnostic information on first occurrence of cardiovascular outcomes covering the period from the start of the trial until December 31, 2009, and obtained from public registers.

##### Demographic and anamnestic data

Clinical data were obtained during enrolment interviews (smoking status, current medication, and known hypertension or diabetes), while information concerning sex, age, and history of myocardial infarction or unstable angina pectoris were obtained from the local hospital files.

##### Biochemical measurements on plasma collected at entry

Blood samples on trial participants were obtained just before randomisation and stored at −80° C. The following quantities were later measured: lipoproteins (total cholesterol, HDL cholesterol, LDL cholesterol, triglycerides, apoprotein A1, apoprotein B), high-sensitivity c-reactive protein (hs-CRP) [15], YKL-40 [16], high-sensitive assay cardiac troponin T (hs-cTnT) [17], pregnancy associated plasma protein-A (PAPP-A) [18], N-terminal pro-B-type natriuretic peptide [17], cathepsin B [19], endostatin [20], cathepsin S [21], soluble TNF receptor 1 and 2 (sTNFR1 and sTNFR2) [22], neutrophil gelatinase-associated lipocalin (NGAL) [23], calprotectin [24], osteoprotegerin [25], and glomerular filtration rate using creatinine (GFR) [26].

##### Vital data and diagnostic information on cardiovascular and other vascular outcomes

Vital status was monitored via the Danish Central Civil Register. Information about the underlying cause of death was obtained from the National Register of Causes of Death (RCD) [27]. The Danish National Patient Register (NPR), covering all somatic hospital admissions, provided hospitalisation data [28]. A blinded adjudication committee, consisting of three cardiologists, working in randomised rotation, assessed each hospital admission or death, using standard diagnostic criteria during the first 2.6 years of follow-up [10, 29]. These results were later compared to the results obtained when only registry data were used and similar conclusions were reached [29, 30]. The follow-up period was therefore extended to 10 years, and the analysis of the full 10 years period was solely based on registry data [10], as explained in the following.

The Danish 10-digit central person registration (CPR) number is used at all contacts with the health care system. Somatic hospital contact cannot be completed without a diagnosis based upon the International Statistical Classification of Diseases, 10th revision (ICD-10) and subsequent notification of the NPR. Each department must issue at least an action diagnosis (A diagnosis), describing the main reason for the admission. Other important diagnoses may be recorded as B diagnoses. All registers have coverage close to 100%. Based on this material, we used the following algorithm to transform the registry information into CV outcomes: for each A code in discharge notifications and, in case of death, each ‘underlying cause of death’ code (in the official terminology of the Registry of Causes of Death), we classified the outcome according to the ICD-10 coding system into a list of disjoint and exhaustive categories: acute myocardial infarction (AMI) (I21.0–23.9), unstable angina pectoris (UAP) (I20.0, I24.8–24.9), cerebro-vascular disease (CeVD) (I60.0–64.9 and G45.0–46.8), peripheral vascular disease (PVD) (I70.2–70.9), and non-cardiovascular disease (A00.0–T98.3 except the codes already covered).

For each patient and each of the outcomes AMI, UAP, CeVD, CV death, and all-cause mortality, we scanned the discharge codes in chronological order. We began with the codes of the first admission following randomisation and recorded the period from the date of randomisation until the date of the first occurrence of the outcome or (in case of a non-fatal outcome) until the date of death or until the date of censoring (December 31, 2009) whichever came first.

##### Classification of the predictors

We have chosen collectively to classify as ‘standard predictors’ the below mentioned groups of predictors, i.e. (1) clinical predictors, (2) current medical treatment, and (3) standard biochemical predictors. The term ‘standard predictors’ is only a collective term used by us in this particular study to refer to those baseline quantities that were available to us during the CLARICOR trial and which are either established prognostic predictors or proxies of such predictors not available to us [31, 32]. The biochemical predictors we have measured in addition for the present PREMAC study are labelled ‘advanced biochemical predictors’. The two groups of predictors, i.e. ‘standard predictors’ and ‘advanced biochemical predictors’ are defined below.

I. Standard predictors
  1. Clinical predictors
    Sex, age, smoking history, history of myocardial infarction compared to angina only, hypertension, and diabetes.
  2. Current medical treatment
    Aspirin (Yes/No), beta-blocker (Yes/No), calcium-antagonist (Yes/No), ACE-inhibitor (Yes/No), long lasting nitrate (Yes/No), diuretic (Yes/No), digoxin (Yes/No), statin (Yes/No), and anti-arrhythmic drugs (Yes/No). The current medical treatment was included as proxy predictors because information about post-infarction heart failure and post-infarction angina pectoris are not available to us.
  3. Standard biochemical predictors
    High-sensitivity-reactive protein, glomerular filtration rate (GFR) estimated by creatinine, and lipoproteins (total cholesterol, HDL cholesterol, LDL cholesterol, triglycerides, apoprotein A1, and apoprotein B).
II. Advanced biochemical predictors
  N-terminal pro-B-type natriuretic peptide, YKL-40, osteoprotegerin, hs-cTnT, PAPP-A, cathepsin B, cathepsin S, sTNFR1, sTNFR2, NGAL, endostatin, and calprotectin. Tests for all these quantities are commercially available.

### Background information on the advanced biochemical predictors

Patients with coronary artery disease (CAD) are usually only evaluated using clinical variables. But some CAD patients have a high incidence of CV insults which are difficult to predict [10]. Measurements of biomarkers may potentially help identifying CAD patients at high risk of such CV insults.

Previously, we found that increased serum N-terminal pro-B-type natriuretic peptide (NT-pro-BNP), a marker of left ventricular dysfunction, and heart failure was a stronger predictor of myocardial infarction (MI), CV death, and non-cardiovascular death than high-sensitive assay C-reactive protein (hs-CRP) in patients with CAD during a 2.6 year follow up [15]. Similar results were found for YKL-40 in the same CAD patients [16]. In this context, it is of pathogenetic interest that YKL-40 is expressed by arteriosclerotic plaque macrophages, particularly macrophages which have infiltrated into the lesion [33]. The highest expression of YKL-40 is found in macrophages in the early atherosclerotic lesion. Another promising marker is high-sensitive assay cardiac troponin T (hs-cTnT) indicating myocardial ischaemia which when combined with the NT-pro-BNP results was found to be significantly associated with all-cause mortality, CV death, and MI after adjustment for traditional risk factors and NT-pro-BNP [17]. Pregnancy-associated plasma protein-A (PAPP-A), a marker of vulnerable plaques in coronary arteries, has also been found to be predictive of CV insults and death in CAD patients [18]. The glycoprotein osteoprotegerin (OPG), which is positively related to coronary calcification, vascular stiffness, and the presence of unstable atherosclerotic plaques [34], is included among the candidate predictors because we found it to be an independent predictor of mortality in CAD patients [25]. The cathepsins are a group of proteinases that have been suggested to be causally involved in the different stages of the atherosclerotic process, from the early stages such as foam cell formation [35] to the later stages, such as destabilisation of the fibrous cap [36]. Endostatin is an endogenous angiogenesis inhibitor where circulating levels have been suggested to mirror an increased neovascularisation induced by vascular or myocardial ischaemia [37], but endostatin has also been suggested to be a marker for an increased extracellular matrix remodelling [38]. Inflammation is a key underlying factor in the atherosclerotic process [39] and tumour necrosis factor receptor alpha TNF-α, and its soluble receptors sTNFR1 and sTNFR2 are inflammatory markers that have been suggested to portray information about a systemic inflammatory state that is independent of other more established inflammatory markers such as CRP or IL-6 [40]. Previous studies report higher levels of cathepsins, endostatin, and TNF-receptors in patients with atherosclerosis and/or CAD [41–43], but whether the proteins are relevant risk markers in these patients remains to be established. Calprotectin, neutrophil gelatinase-associated lipocalin (NGAL), and myeloperoxidase are all released from neutrophils when the cells are activated. Circulating levels of neutrophils and their activation products have been shown to be markers for plaque instability in both primary and secondary prevention of cardiovascular diseases [44, 45]. We have also previously shown that U-NGAL is associated with cardiovascular mortality [23]. We now aim to explore if calprotectin is a risk marker in these patients.

### Statistical analysis

Each of the abovementioned advanced biochemical predictors will be studied individually on the below mentioned outcomes provided that the advanced quantity concerned has shown promise in the primary (‘gatekeeper’) analysis. This primary ‘gatekeeper’ analysis will examine a composite outcome, defined to be present if at least one of the events AMI, UAP, CeVD, and all-cause death has occurred in a patient. The analyses will be conducted using the Cox proportional hazard model supplemented by Breslow estimation of the baseline hazard. We will use SAS 9.4, and the analysis will be based on the data from the placebo group because it was previously shown that clarithromycin had a significant and harmful effect on the prognosis of the trial patients [10, 12–14].

#### The proportional hazards assumption

The joint proportional hazard property, covering all covariates included in a Cox analysis and the chosen functional forms of quantitative covariates, will be tested using cumulative sums of martingale-based residuals over follow-up time and/or covariate values [46]. The test statistic is a Kolmogorow-type supremum test, using for each outcome a *P* threshold = 0.05 and Bonferroni adjusted *P* values.

#### Flow of analyses

For each advanced biochemical quantity, we will examine if the hazard ratio of the biochemical quantity is different from 1.00 with a *P* value <0.01 when used alone and when used in combination with the standard predictors (forced to be included in the analyses whether they are significant or not). Using centre ID as a class variable, we will stratify on centre ID implying that the observations are conditionally independent within centres and that the coefficients of the covariates are the same across centres.

For each outcome, we will test for interaction between sex and each clinical predictor, each standard biochemical predictor, and each advanced biochemical predictor using Bonferroni adjusted *P* values and 0.05 as a threshold. The interactions corresponding to adjusted *P* values below 0.05 will be included in the model. Explanation will be sought for any marked discrepancies between the two settings (candidate used alone and candidate used in combination with standard predictors); but we will let us be guided by the results obtained when the latter setting is used.

Those advanced biochemical quantities which have a hazard ratio different from 1.00 and a *P* value <0.01 when used in combination with the standard predictors in the initial ‘gatekeeper’ analysis, will be included in subsequent and similar analyses of each individual outcome of the composite outcome.

We will use the *P* values of the analyses as a data-reducing device where a threshold of *P* < 0.01 is used at each decision node on our way to identify a candidate biochemical predictor among the advanced biochemical predictors. We regard the set of selected advanced biochemical predictors as the primary result of this essentially hypothesis-generating effort. However, clinically implausible results will be discussed and commented on.

#### Illustration of predictive impact

Nine, six and three-year estimated incident risks will be calculated for each patient using the Cox-Breslow procedure. We will illustrate discrimination by the reclassification indices, reported separately for those with and those without the event in question as recommended by Kerr et al. [47]. For example the risk estimates could be categorised as less than 25% (‘negative’) or 25% or more (‘positives’). With only two categories, this amounts to reporting the changes in the true-positive and false-positive rates.

For the mortality models, we present measures of risk differentiation, obtained by imagining preventive measures administered under budget constraints. The C statistic (Harrell’s concordance statistic) [48] represents the proportion of all patient pairs where the predicted survival is better for the patient who survived longer. The fixed budget constraint counts ‘lives saveable [by a hypothetical intervention in high-risk patients]’ [25].

#### Missing values

There are virtually no missing data from entry information in the CLARICOR trial [10, 12]. We expect missing laboratory data to be MCAR due to the fact that we know that some serum samples are missing either due to missing blood sample in the first place or due to a vial being damaged (see discussion). We will use Little’s test to decide whether a multiple imputation or a complete case analysis should be conducted [49]. Thus, each analysis of an advanced predictor will be initiated by Little’s test which will include all covariates with missing values (the standard biochemical predictors plus the advanced biochemical predictor to be assessed) plus all other variables in the model.

#### Supplementary exploration

Additional supplementary analyses include (1) an analysis of the impact of alternative GFR definitions and (2) an assessment of whether chance imbalance of new biochemical predictors may dramatically change the original findings of the CLARICOR trial.

The creatinine-based GFR will be used as our quantitative measure of kidney function in this study. Within the framework of the model that we will develop, we will in a separate report compare the predictive power of different formulas to estimate GFR (creatinine-based GFR, cystatin C-based GFR, or a combined creatinine/cystatin GFR formula). We will divide participants in GFR categories (GFR >60, 30–60, and <30 mL/min/1.73 m^2^) according to each specific GFR equation. We will then study the impact of reclassification across the GFR categories by the different GFR equations, by assessing the risk of adverse outcomes in those who were reclassified to a higher GFR category or to a lower GFR category, compared to those who were not reclassified.

Finally, we will assess if skewness (chance imbalance) in any predictors may explain the increased cardiovascular and all-cause mortality in participants who received clarithromycin as compared with placebo participants, i.e. we will repeat the analysis of the effect of clarithromycin but this time include the biochemical prognostic markers identified in the above described study.

## Discussion

The present PREMAC study is important mainly for two reasons. First, new prognostic indicators are needed to improve preventive interventions in patients with ischaemic heart disease. Second, if known or new prognostic indicators show skewness between the two intervention groups at entry into the CLARICOR trial, this may help us better understand the harmful effects of clarithromycin. However, in previous publications we found no skewness of hs-CRP [15], N-terminal-pro-B-type natriuretic peptide [17], PAPP-A [18], YKL-40 [16], osteoprotegerin [25], and hs-cTnT [17].

The strengths of the CLARICOR trial are the considerable size of the patient population, long duration of follow-up, very few losses to follow-up (0.5%), the ethnic homogeneity of the patient population, and rarity of missing values. However, the values of 20 biochemical quantities have been added to the CLARICOR data and these data have missing values. The set of placebo patients with one or more missing biochemical quantity values includes 210 patients out of 2199 placebo treated patients giving 210/2199 = 9.5% patients having one or more biochemical quantity values missing. The percentage missing for the individual biochemical quantities ranges between 1.6 and 5.7%.

Potential weaknesses of the present cohort include the lack of information about left ventricle function, body mass index, blood pressure, and changes in medications during the follow-up period, and the fact that the prognosis of this type of patients today may have changed somewhat from the year 2000. Information about post-infarction heart failure and post-infarction angina pectoris was not available to us. Therefore, we added information about the medication at entry into CLARICOR as proxy information instead. The lack of information about left ventricular ejection fraction may be partially or completely compensated as Solomon et al. [32] found that age, sex, hypertension, prior AMI, creatinine, diuretics, digoxin, and mineralocorticoid receptor antagonist were related to left ventricular ejection fraction, all quantities that we have included within the group referred to as ‘standard predictors’.

Selection bias is also a potential weakness of the study. Only 2.6% of the patients selected for the CLARICOR trial refused to participate. So far that problem seems negligible. However, among those 7586 patients who declined our invitation to visit a cardiology centre, many must have been eligible for the CLARICOR trial, and we do not know how they looked and fared. So selection bias is a possibility.

The design of the CLARICOR trial may also contribute to selection bias. In the CLARICOR trial, the rate of cardiovascular events and deaths was studied in patients who had contracted an AMI or UAP during the period January 1993 to August 1999, were alive during the randomisation period (October 1999 to April 2000) and fulfilled the inclusion criteria and none of the exclusion criteria as defined in the CLARICOR trial [10].

Normally, to assess the rates in stable CAD patients, one would have started the study in January 1993 by including consecutively each patient who contracted an AMI or UAP, monitor the patient at regular intervals, say monthly from then on, note when the patient fulfilled the stability criteria (i.e. entered the stable state) and when the patient left the stable state again, and noting the characteristics of the new state (e.g. death). The patients included in such a design would differ from those of the CLARICOR trial in that they would include patients who entered the stable state and then died before October 1999. These patients are missing in the present PREMAC study. Thus, using the data to predict the prognosis of patients with stable CAD (as defined by the CLARICOR group), the results may very well be biased. This is the reason why we emphasise that the PREMAC study is an exploratory and hypothesis generating study.

We confined the net reclassification indices to the increase in the true-positive rate and the decrease in false-positives by including only two risk groups because when there are more than two risk categories, the indices do not adequately account for clinically important differences in shifts among risk categories [47]. We did not use category-free net classification indices because they suffer from many of the same problems as similar measures such as, e.g. the area under the receiver operating characteristic curve does. Besides, it can mislead investigators by overstating the incremental value of a biomarker, even in independent data [50].

A single number summary of the prediction increment would be the improvement in net benefit. This would require us to know the ratio between the cost of falsely classifying an event as a non-event and the cost of classifying a non-event as an event. (If this ratio was 3:1, it would dictate a predicted risk of 25% as a decision threshold; the cut at 25% mentioned above was foreseen to be an illustrative cut and was not suggested to be a clinical decision threshold.) We do not know which value of this ratio will be appropriate; anyhow, we may decide to adapt the Vickers-Elkin net benefit diagram [51] to the PREMAC context.

Clearly, the prognosis for the occurrence of all-cause mortality or CV mortality as predicted by the baseline values may very well change once it becomes known that one of the non-fatal vascular outcomes has occurred. Thus, this valuable outcome information should be studied not only in its role as being an outcome as described above but also in its role as being a predictor of all-cause mortality and CV mortality. We plan to analyse these time-series aspects in a subsequent study using intervening MI and UAP as predictive events.

## Abbreviations

AMI: Acute myocardial infarction
CeVD: Cerebro-vascular disease
CLARICOR: Clarithromycin for patients with stable coronary heart disease
CPR: Central personal registration number
CV: Cardiovascular
GFR: Glomerular filtration rate
hs-cTnT: High-sensitive assay cardiac troponin T
ICD: International Statistical Classification of Diseases
NGAL: Neutrophil gelatinase-associated lipocalin
NPR: National Patient Register
NYHA: New York Heart Association
PAPP-A: Pregnancy-associated plasma protein-A
PREMAC: Predictors for major cardiovascular outcomes in stable ischaemic heart disease
PROGRESS: Prognosis Research Strategy
RCD: Register of Causes of Death
STNFR1: Soluble TNF receptor 1
STNFR2: Soluble TNF receptor 2
UAP: Unstable angina pectoris
YKL-40: A 40 kDa heparin- and chitin-binding glycoprotein also known as human cartilage glycoprotein 39
